# Fluoroquinolone-induced generalised fixed drug eruption: a rare clinical image

**DOI:** 10.11604/pamj.2025.50.56.45087

**Published:** 2025-02-18

**Authors:** Sreeprada Bollineni, Gaurang Aurangabadkar

**Affiliations:** 1 Department of Respiratory Medicine, Jawaharlal Nehru Medical College, Datta Meghe Institute of Higher Education and Research (DMIHER), Deemed University, Sawangi (Meghe), Wardha, India,; 2 Department of Respiratory Medicine, Datta Meghe Medical College, Nagpur, India,; 3 Datta Meghe Institute of Higher Education and Research (DMIHER), Deemed University, Sawangi (Meghe), Wardha, India

**Keywords:** fluoroquinolones, atypical pneumonia, drug eruptions

## Image in medicine

Fluoroquinolones are commonly used as antimicrobial agents in patients presenting with respiratory diseases with levofloxacin, ofloxacin, and moxifloxacin, and have a significant role in the management of pulmonary tuberculosis and atypical pneumonia. However, the use of fluoroquinolones is also associated with the development of adverse drug reactions such as tendon rupture, Qtc interval prolongation, and generalised drug eruptions. Here, we present a 70-year-old male patient who presented with generalised drug eruption rash over the body 3 days after starting levofloxacin therapy for atypical pneumonia. The patient was treated as per the dermatologist's advice with oral corticosteroids and antihistamines. After 7 days, significant improvement was noted in skin eruptions. This clinical image aims to highlight a rare adverse effect of a commonly used antimicrobial agent.

**Figure 1:**
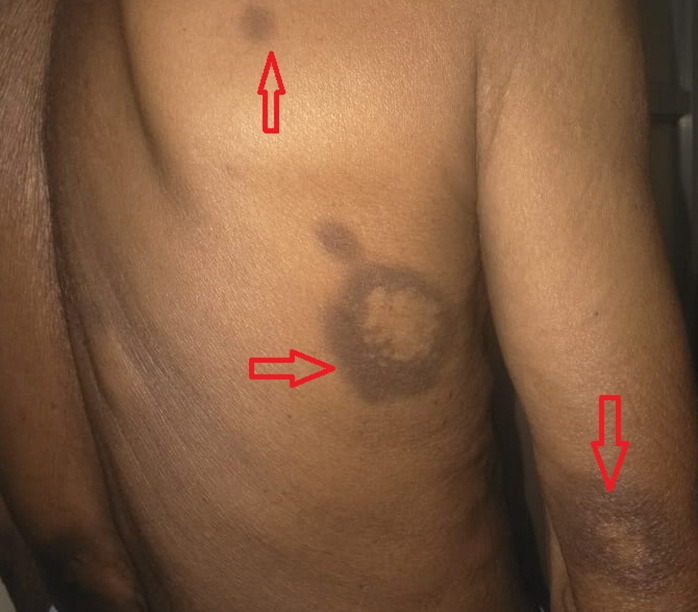
multiple lesions of generalised drug eruption in a patient treated with levofloxacin

